# Impact of Patient Engagement on Blood Pressure Control Among Older Individuals With Hypertension in a Mobile Health Intervention: Longitudinal Analysis Using Latent Growth Curve Modeling

**DOI:** 10.2196/71668

**Published:** 2025-07-31

**Authors:** Nanxiang Zhang, Hai Lin, Xichun Wu, Yongjun Zheng, Jianan Yin, Chonglong Ding, Qi Pan, Shuo Yang, Hao Luo, Xinyan Zou, Yingfeng Ge, Jinxin Zhang

**Affiliations:** 1 Department of Medical Statistics, School of Public Health, Sun Yat-sen University, #74 Zhongshan 2nd Road, Guangzhou, China, 86 13660501365; 2 Zhongshan Center for Disease Control and Prevention, Zhongshan, China; 3 Zhongshan Rehabilitation Hospital, Zhongshan, China; 4 Sanxiang Community Health Service Center of Zhongshan, Zhongshan, China

**Keywords:** mHealth, patient engagement, hypertension, latent growth curve model, aging population, mobile health

## Abstract

**Background:**

Limited research has investigated the influence of patient engagement on the long-term effects of mobile health (mHealth) interventions, particularly among older adults.

**Objective:**

This study aimed to examine the long-term impact of a social media–driven mHealth intervention on blood pressure control among older Chinese individuals with hypertension, through repeated measurements of patient engagement and outcomes at 5 preset time points.

**Methods:**

The study included older Chinese individuals with hypertension between 2017 and 2022. Participants received a hypertension self-management program via the WeChat social media app (Tencent Holdings Ltd), which provided clinically based digital coaching. Blood pressure measurements were taken repeatedly using a home blood pressure monitor (HBPM) connected to the app at baseline, 3, 6, 9, and 12 months. Patient engagement was evaluated based on the frequency of completed measurements at corresponding follow-ups. Latent growth curve models (LGCMs) served to assess the impact of patient engagement on blood pressure among older individuals with hypertension across preset points.

**Results:**

A total of 1723 patients completed the 12-month follow-up (average age 70.1, SD 6.8 years; 890/1723, 51.7% female; and baseline systolic blood pressure 137.2 mm Hg). LGCMs revealed systolic blood pressure decreased significantly over 1 year, notably at 9 months (131 mm Hg, β_9_=3.244, *P*<.001), and continued up to 12 months (131.6mm Hg, β_12_=2.827, *P*<.001). In addition, a higher frequency of completed measurements was associated with better systolic blood pressure control at 3, 6, 9, and 12 months (β_3_=–0.016, *P*=.002; β_6_=–0.006, *P*=.02; β_9_=–0.002, *P*=.44; β_12_=–0.003, *P*=.02). These results remained significant even after accounting for age, sex, and comorbidity status.

**Conclusions:**

This study, using LGCMs and repeated measures data, revealed a significant positive impact of patient engagement on long-term blood pressure control in mHealth interventions targeting older individuals with hypertension. These findings stress the importance of integration of patient-centered engagement approach into mHealth programs designed for chronic disease management in aging populations.

## Introduction

Noncommunicable diseases, often referred to as chronic diseases, are a main cause of disability and death globally [1]. The aging population, coupled with unhealthy behaviors such as sedentary habits and unbalanced diet, led to a dramatic increase in chronic disease risk factors, including hypertension [2,3]. Hypertension is a chronic condition that progresses gradually over time, requiring continuous care and self-management [4,5]. Health care services based on mobile health (mHealth) are considered to be beneficial for older individuals with hypertension as they facilitate a range of favorable outcomes, including health care cost-effectiveness, personalized medical care, and enhanced feedback on health information [6]. Thus, mHealth holds the promise of enhancing the quality and longevity of life for older adults through the transformation of health care delivery and personalized clinical support [7].

Older adults can potentially gain significant benefits from using mHealth services. However, the association between patient engagement and the results of mHealth services among older patients remains unclear [8,9]. Historically, patient engagement has been characterized by how proactively participants engage with the intervention [10]. In traditional face-to-face studies, patient engagement was viewed as a crucial factor influencing health improvement. Increased patient participation is linked to better intervention effectiveness [11]. However, this relationship remains unconfirmed in the context of mHealth interventions [12]. In the context of mHealth interventions for older adults, results regarding the relationship between patient engagement and intervention effectiveness have been inconsistent. Several studies have uncovered relationships resembling those observed in traditional interventions [13-15], whereas other research has not yet identified significant relationships [16-18]. Some studies found that patient engagement was a dynamic and ongoing process [19]. For instance, some patients registered but did not start the app or used it intermittently, while others used it frequently over the long term [20]. These different activities of using the app may make the results of the intervention different. Consequently, it is necessary to further investigate how patient engagement affects health outcomes to clarify the dose-response relationship within interventions.

Few mHealth studies in program evaluation have explored the dose-response relationship. Among the limited studies, the most used is the pre-post design with a brief follow-up period (typically within 12 weeks) [14-16,18]. A significant gap exists in the continuous assessment of this relationship between patient engagement and health improvements throughout the intervention period. It remains uncertain whether mHealth interventions yield sustained benefits. Specifically, it is not clear whether this relationship emerges early (like traditional interventions) [21] or later. Current mHealth studies differ in intervention lengths, potentially resulting in varied findings concerning this relationship. Among mHealth interventions targeting blood pressure control in the study population, some lasted 2 weeks, while others extended to 6 months [13,15]. The diverse durations of mHealth interventions complicate the comparison of dose-response relationships. This challenge is particularly pronounced when the relationship is assessed only at pre- and postintervention time points. Long-term mHealth research is warranted to gain a clearer understanding of the potential relationship between patient engagement and intervention effectiveness [22-24]. Longitudinal studies with multiple measurements (at least 3) may enable the assessment of the time-varying impact of patient engagement on health improvements [25].

Inspired by the aforementioned research, this study makes several unique contributions to the existing literature. First, the study explored the relationship between patient engagement and changes in blood pressure over a 1-year follow-up period in an mHealth program targeting older individuals with hypertension. By extending the time period, we can more fully assess how patient engagement has evolved over time and evaluate its sustained impact on blood pressure control. Second, guided by insights from recent systematic reviews of adherence studies, we used a reliable indicator to assess various dimensions of longitudinal relationships [26,27]. We assessed patient engagement and blood pressure levels, as well as their changes, at 3, 6, 9, and 12 months, using outcome and engagement measures across 5 time points. Third, this study adopts a more refined longitudinal modeling approach, latent growth curve models (LGCMs), to explore their dose-response relationship at these intervals. We hypothesize that greater engagement in long-term mHealth interventions might contribute to more favorable and enduring health outcomes.

## Methods

### Study Design

The study is a cohort investigation of individuals using a hypertension self-management program adapted from the “National Basic Public Health Service Standards (Third Edition, 2017)” [28]. The study sample comprised managed care hypertension cases from 50 primary care communities of Zhongshan City, Guangdong Province, China, from January 1, 2017, to December 31, 2022.

### Description of the mHealth Intervention

**Figure 1.**
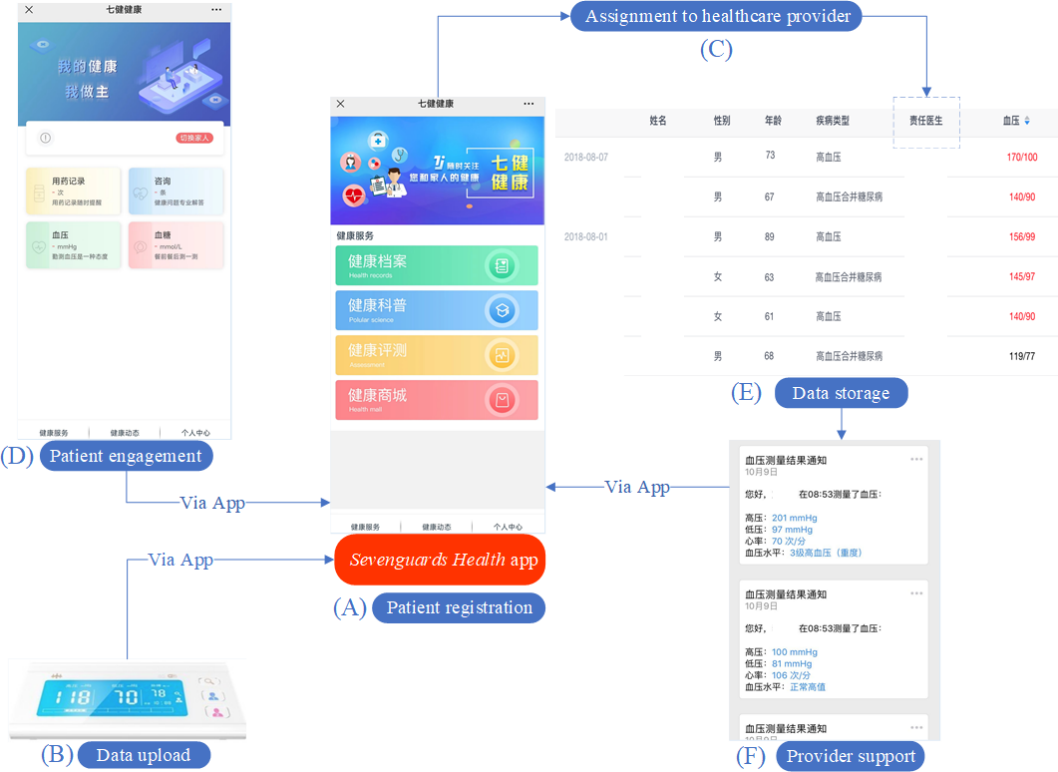
The diagram of the Sevenguards Health app. (**A**) Patient registration: after signing up for the Sevenguards Health app, patients input key demographic details like name, sex, date of birth, and comorbidity status. (**B**) Data upload: patients track their health by uploading data such as blood pressure readings, medication logs, physical activity records, and dietary information. (**C**) Assignment to health care provider: each patient is linked with a health care provider and receives a personalized management plan. (**D**) Patient engagement: patients use the app to track blood pressure and plan adherence. (**E**) Data storage: all collected data are securely stored on the Sevenguards server. (**F**) Provider support: providers monitor data and use WeChat to communicate, conduct follow-ups, and assist in blood pressure control.

The mHealth intervention described here is a comprehensive digital health program aimed at improving hypertension management among patients. It uses the Sevenguards Health app, which serves as a user-friendly mobile app and a supportive digital platform for self-management and engagement between patients and health care providers (Figure 1).

This diagram provides an overall interface of the Sevenguards Health app and guides users through its entire process. Patients register and provide key demographic details (Figure 1A), then upload data from HBPMs to the connected app (Figure 1B). They are assigned to specific health care providers (Figure 1C) and engage in self-management via the app (Figure 1D). The Sevenguards server handles data storage and management (Figure 1E). The app informs providers of patients’ conditions, supporting follow-up management (Figure 1F).

### Patients

Community doctors first screened patients from their villages who might be eligible and invited them to join the study. Subsequently, the research team completed the final recruitment and obtained consent from participants. These participants were then further enrolled (N=3251) into the mHealth hypertension management program. The main inclusion criteria were participants who (1) were aged 60 years or older, (2) had a documented history of hypertension from county-level or higher-level hospitals, and (3) possessed at least basic communication skills. Patients who were bedridden, had severe life-threatening conditions, or had a life expectancy of less than 6 months were excluded from the study. To promote inclusivity, neither mobile phone ownership nor technological literacy was a criterion for patient recruitment.

The primary dataset comprised 1723 patients. Patient records were selected based on registration with the Sevenguards Health app between January 1, 2017, and December 31, 2022. During this period, (1) the app’s core functions remained stable, ensuring that patient behavior was not affected by changes in functionality; and (2) blood pressure was continuously measured and recorded for over 12 months following registration.

### Measurement

#### Blood Pressure

To address potential measurement bias, we implemented several quality control measures for blood pressure measurements. The Lexin i5/i7 HBPMs were used for measurements. Before being issued to participants, all HBPMs were calibrated according to manufacturer guidelines. Participants measured their blood pressure using the built-in cellular-connected HBPMs, which are connected to the Sevenguards Health account and require no additional setup.

During the study period, participants received standardized training from the community doctors. This training ensured the consistency and standardization of home blood pressure measurement methods in line with the guidelines issued by the China Blood Pressure Measurement Working Group in 2011 [29]. For both underlying measurement and psychological reasons [30], blood pressure values measured on day 1 tend to be slightly higher than those from subsequent days. Many guidelines, therefore, advise against using day 1 measurements to guarantee a more accurate blood pressure reading [31]. Accordingly, following the initial guidance, we used second-day readings as the baseline to mitigate issues with initial measurements. Ongoing support via WeChat (Tencent) was provided to ensure participants maintained proper measurement practices.

To assess blood pressure over time, we calculated (1) the mean blood pressure at specific time points: baseline (day 2, representing the initial blood pressure level in hypertension management) and (2) follow-up intervals, assessing the monthly mean blood pressure trends at 3 (range 0‐3) months, 6 (range 4‐6) months, 9 (range 7‐9) months, and 12 (range 10‐12) months from the baseline. Since participants joined the program at different times, the follow-up duration varied among them. Data beyond the 12-month report were not analyzed.

#### Patient Engagement

Patient engagement was assessed using the frequency of completed measurements for several reasons. Most mHealth studies on blood pressure management rely on apps or Bluetooth-connected blood pressure devices. In related studies, measurement frequency has been widely used for its simplicity and directness [32]. Clinical guidelines also emphasize that HBPM must meet a certain frequency to ensure the reliability and effectiveness of blood pressure management [33]. Thus, the frequency of completed measurements was considered a reliable indicator for engagement in mHealth interventions targeting health outcomes. The indicator reflected how often a participant used the HBPM during the specified blood pressure measurement periods. For instance, if a participant measured his blood pressure twice during the 0- to 3-month intervention period, the frequency of completed measurements would be recorded as 2. Since participants were encouraged to measure their blood pressure multiple times, this indicator was used to reflect repetitive feature of patient engagement. Both blood pressure measurements and measurement frequency were automatically recorded by the app.

#### Baseline Characteristics

Demographic data encompassed age, sex, and comorbidity status. Comorbidities, including diabetes, were self-reported by participants within the mHealth app.

### Statistical Analysis

Descriptive statistics on blood pressure, patient engagement, and baseline characteristics were provided. Continuous variables that followed a normal distribution were summarized using means and SDs. Nonnormally distributed variables were characterized by medians and interquartile ranges. Categorical variables were reported as counts and proportions.

LGCMs were used to assess the impact of patient engagement on blood pressure. This method was specifically chosen due to its unique advantages in analyzing longitudinal data with repeated measurements [34]. LGCMs are particularly well suited to capture both the intercept and slope of blood pressure changes over time, as well as the time-varying effects of patient engagement. They provide a comprehensive understanding of the dynamic relationship between patient engagement and blood pressure control, which is crucial for evaluating the long-term effects of the mHealth intervention. Unlike traditional repeated measures ANOVA, LGCMs can accommodate nonlinear patterns of change and handle missing data more flexibly, which is particularly useful in longitudinal studies where participants may miss scheduled assessments. In addition, LGCMs enable the integration of time-invariant and time-varying covariates, allowing us to control for demographic characteristics while investigating the dynamic relationship between patient engagement and blood pressure.

Robust maximum likelihood estimation served to address missing data and to estimate model parameters [34]. Descriptive analyses were performed in R (v 4.4; R Core Team). LGCMs were executed using Mplus v 7.0 (Muthén & Muthén). The above-mentioned analyses were performed at the individual level using an intention-to-treat framework.

The LGCM analysis was conducted in 2 steps [34]. Given the strong correlation between diastolic and systolic blood pressure, only the latter was included in LGCMs [20]. First, an unconditional LGCM was developed to model temporal changes in blood pressure. The baseline blood pressure was modeled as the intercept, with the average temporal change represented as the slope. Loadings for the intercept factor were uniformly set to 1. In growth curve modeling, the slope factor is key to summarizing the rate of change over time. The first loading is fixed at 0, serving as a baseline reference point with no change. The second loading is fixed at 1, indicating a unit change from the first to the second timepoint. The remaining loadings are objectively estimated from the data. If growth is linear, with a consistent rate of increase of 1 unit per timepoint, the loadings would ideally be 0, 1, 2, 3, and so on, representing a linear increase. However, if the growth is nonlinear, the loadings for later time points will significantly differ from those aforementioned integer values. For example, if the third loading is estimated to be 1.5 and the fourth loading is 2.8, this suggests a nonconstant rate of change. The deviation from the expected linear values of 2 and 3 indicates a more complex growth trajectory. This method of fixing the first 2 loadings and freely estimating the rest allows researchers to test the assumption of linearity and identify any deviations from a linear growth pattern, providing valuable insights into the underlying dynamics of the growth process.

Second, a conditional LGCM was developed to investigate how patient engagement influences blood pressure. It was an extension of unconditional LGCM, incorporating patient engagement and demographic characteristics as control variables. The relationship between patient engagement and health outcome was modeled as a time-varying effect. Specifically, health outcome (eg, systolic blood pressure at 3 months) was regressed on the corresponding patient engagement metric (eg, completion frequency from 0 to 3 months). Thus, patient engagement was regarded as a time-specific predictor of blood pressure. The conclusion was reached after adjusting for demographic characteristics and underlying trajectory of blood pressure.

The LGCM fit was assessed using chi-square tests and several indices, such as the standardized root mean square residual, Tucker-Lewis index, confirmatory fit index, and root mean square error of approximation. Model fit was considered adequate based on the following criteria: Tucker-Lewis index ≥0.95, confirmatory fit index ≥0.95, standardized root mean square residual ≤0.08, and root mean square error of approximation ≤0.08 [35].

### Ethical Considerations

This study received ethical approval from the Public Health School of Sun Yat-sen University (approval number 026/2022) and complied with the Declaration of Helsinki. All participants were fully informed about the study procedures and objectives, after which they voluntarily agreed to take part and provided written consent. Participants were offered free use of HBPMs, but no additional financial or material incentives were provided for participation. All data were securely stored on the Sevenguards server. All patient data underwent deidentification processing to ensure anonymity.

## Results

### Baseline Characteristics

Table 1 shows the average age of individuals was 70.1 (SD 6.8) years. Approximately half of the overall sample identified as female, comprising 890 out of 1723 participants (51.7%). In addition, 113 participants out of 1723 (6.6%) reported having both hypertension and diabetes.

**Table 1.** Demographic characteristics of patients (N=1723).

Variables	Value
Age (years), mean (SD)	70.1 (6.8)
Sex, n (%)	
Male	833 (48.3)
Female	890 (51.7)
Comorbidity status, n (%)	
Hypertension without diabetes	1610 (93.4)
Hypertension with diabetes	113 (6.6)

### Change in Blood Pressure and Patient Engagement Over Time

Of the participants, 92.5% (1602/1723), 92.3% (1599/1723), 91.1% (1578/1723), and 92.7% (1606/1723) completed the blood pressure measurements at 3, 6, 9, and 12 months, respectively. Compared with mean systolic blood pressure at baseline (137.2, SD 19 mm Hg), subsequent follow-up assessments showed a reduction in systolic blood pressure. The mean values were 134.1 (SD 13.5) mm Hg, 133.3 (SD 13.8) mm Hg, 131 (SD 13.6) mm Hg, and 131.6 (SD 13.3) mm Hg at 3, 6, 9, and 12 months, respectively (Table 2). The mean diastolic blood pressure was 77.3 (SD 8.8) mm Hg, 77 (SD 9.1) mm Hg, 75.9 (SD 9) mm Hg, and 76 (SD 9) mm Hg at 3, 6, 9, and 12 months, respectively.

**Table 2.** Systolic blood pressure and patient engagement across the 12-month intervention.

Variables^a^	Value	
	Mean (SD)	Median (IQR)
Systolic blood pressure		
Baseline	137.2 (19)	138 (125‐149)
3 months	134.1 (13.5)	133 (125‐142)
6 months	133.3 (13.8)	132 (124‐141)
9 months	131 (13.6)	130 (121‐139)
12 months	131.6 (13.3)	130 (122‐139)
Diastolic blood pressure		
Baseline	79.3 (11.6)	79 (71‐87)
3 months	77.3 (8.8)	77 (71‐83)
6 months	77 (9.1)	76 (70‐83)
9 months	75.9 (9)	75 (69‐81)
12 months	76 (9)	75 (69‐81)
Frequency of completed measurements		
3 months	32.3 (44.6)	18 (8‐40)
6 months	63.7 (88.6)	36 (17‐75)
9 months	64.1 (90.5)	36 (16‐78)
12 months	64.4 (95.1)	34 (16‐76)

a For blood pressure measurement, sample sizes were 1602, 1599, 1578, and 1606 at 3, 6, 9, and 12 months, respectively. For patient engagement, it remained 1723 throughout the 12-month intervention.

**Figure 2.**
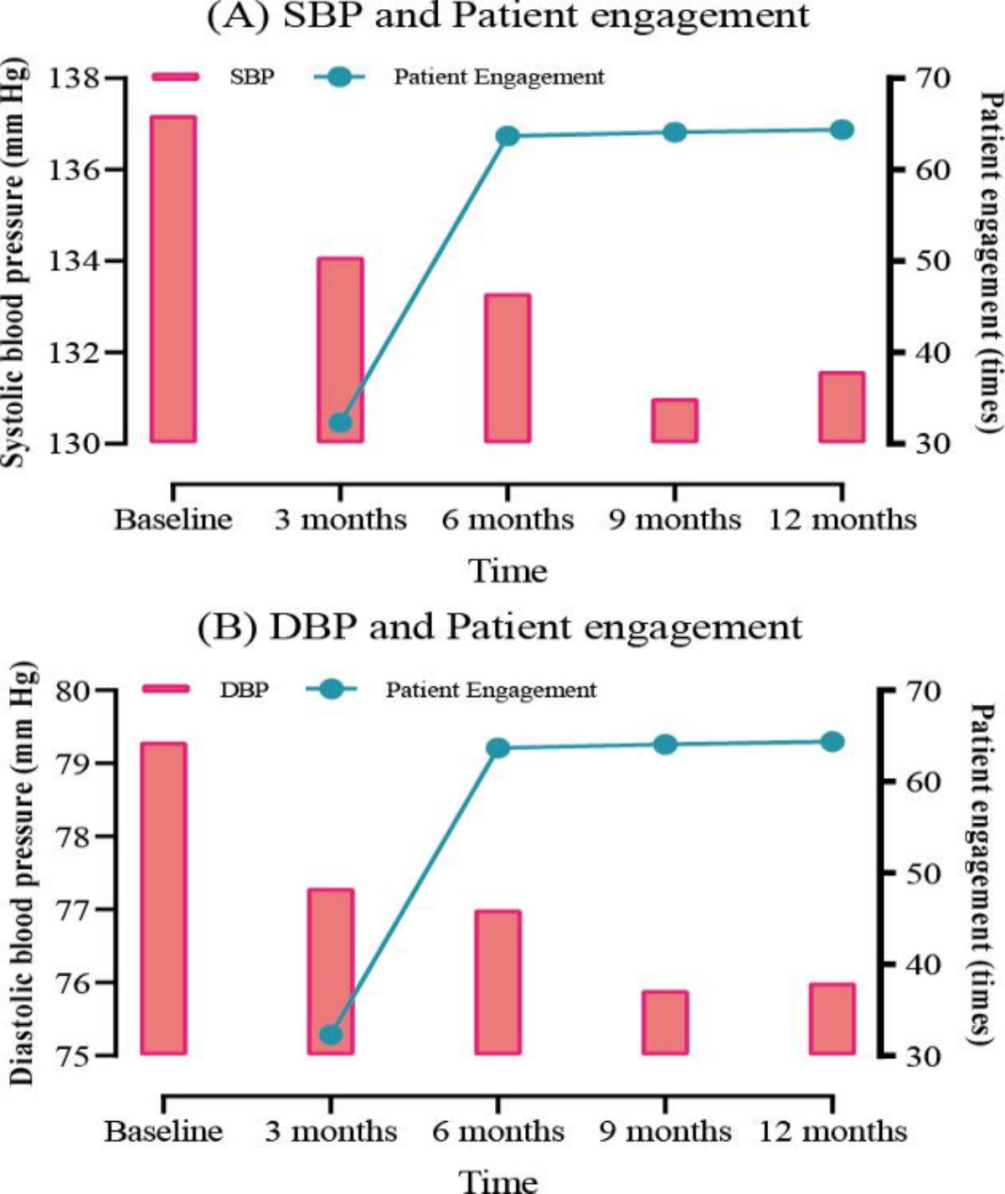
Changes over time in blood pressure and patient engagement. DBP: diastolic blood pressure; SBP: systolic blood pressure.

Approximately 94.4% participants (1627/1723) uploaded blood pressure data to at least 3 time points. The gradual increase in patient engagement was observed throughout the study period. The mean frequencies of completed measurements were 32.3 (SD 44.6) times, 63.7 (SD 88.6) times, and 64.1 (SD 95.1) times at 3-, 6-, 9-, and 12-month follow-ups, respectively. Upon comparing the values in Table 2, the distribution of mean patient engagement was found to be positively skewed. Specifically, mean values were substantially higher than the medians at 3, 6, 9, and 12 months. These trends are visually represented in Figure 2, illustrating the changes in systolic (Figure 2A) and diastolic (Figure 2B) blood pressure alongside patient engagement over the study period.

### Unconditional LGCM

**Figure 3.**
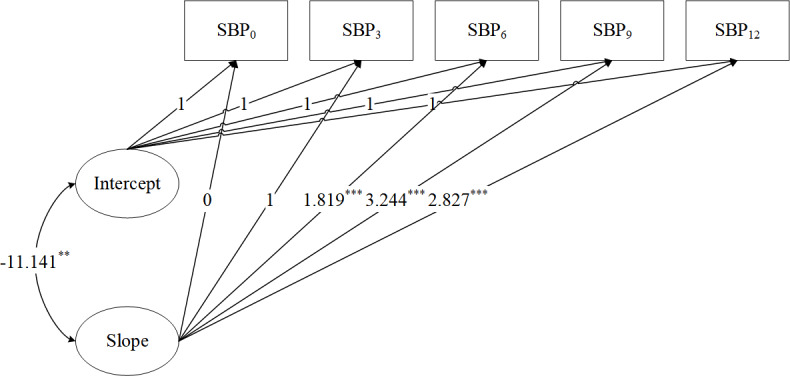
Unconditional latent growth curve model for SBP (N=1723). Model fit statistics were *χ*^2^_7_=89.351 (*P*<.001), Tucker-Lewis index=0.957, confirmatory fit index=0.970, standardized root mean square residual=0.112, and root mean square error of approximation=0.083. Observed variables are depicted with boxes. Latent variables are represented by ovals. Unidirectional arrows show the influence of one variable on another. Bidirectional arrows denote correlations. A dotted line indicates nonsignificant paths. Time points are represented as: 0 (baseline), 3, 6, 9, and 12 months post baseline. **P<.01, ***P<.001. SBP: systolic blood pressure.

The unconditional LGCM, which demonstrated good fit (Figure 3), showed that systolic blood pressure declined throughout the 12-month intervention program, with the most significant reduction occurring at the 9-month timepoint following baseline. The mean intercept value of 136.177 (SD 10.69; *P*<.001) reflects a baseline systolic blood pressure of 136.177 mm Hg and the mean slope value of –1.559 (SD 2.941; *P*<.001). Factor loadings on the slope were 0, 1, 1.819, 3.244, and 2.827 at 5 preset time points. The results suggested a nonlinear trend in systolic blood pressure reduction. There was a rapid decrease in systolic blood pressure (–1.559 units) at 9 months, followed by a plateau in the reduction. The variance of intercept was 114.271 (*P*<.001), suggesting significant individual variability in baseline blood pressure. Meanwhile, the variance of slope was 8.651 (*P*=.02), indicating substantial variability in change rate. The covariance between the 2 was –11.141 (*P*=.003). It indicated a significant inverse correlation between the rate of change in individual systolic blood pressure and the initial level. In other words, individuals with higher baseline systolic blood pressure experienced a slower decline over time (Table 3).

**Table 3.** Estimated parameters for unconditional latent growth curve model for systolic blood pressure (SBP; N=1723).

Growth parameters	Estimate	SE	*P* value
Slope_0_→SBP_0_	0.000	0.000	—^a^
Slope_1_→SBP_3_	1.000	0.000	—^a^
Slope_2_→SBP_6_	1.819	0.261	<.001
Slope_3_→SBP_9_	3.244	0.607	<.001
Slope_4_→SBP_12_	2.827	0.482	<.001
Mean intercept	136.177	0.475	<.001
Mean slope	−1.559	0.400	<.001
Variance of intercept	114.271	7.847	<.001
Variance of slope	8.651	3.657	.02
Covariance of intercept and slope	−11.141	3.758	.003

a Not available.

### Conditional LGCM

**Figure 4.**
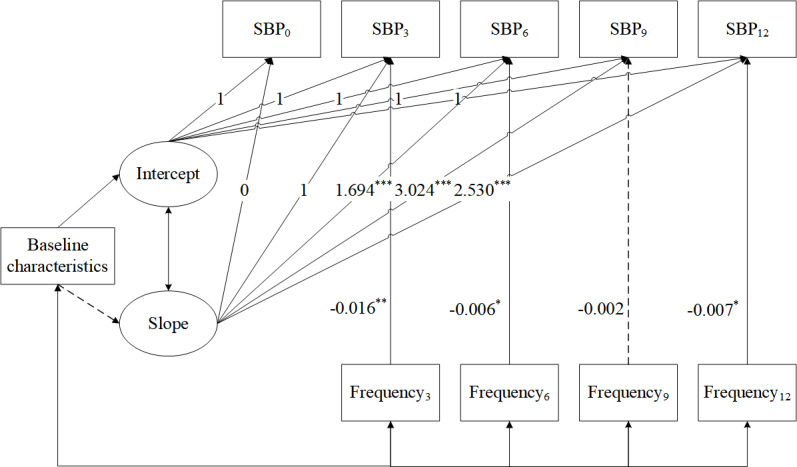
Conditional latent growth curve model for systolic blood pressure (SBP) with patient engagement (N=1723). Model fit statistics were *χ*^2^_32_=121.150 (*P*<.001), Tucker-Lewis index=0.956, confirmatory fit index=0.968, root mean square error of approximation=0.040, and standardized root mean square residual=0.064. Baseline characteristics included age, sex, and comorbidity status. Unidirectional arrows show the influence of one variable on another. Bidirectional arrows denote correlations. A dotted line indicates nonsignificant paths. Time points are represented as: 0 (baseline), 3, 6, 9, and 12 months post baseline. *P<.05, **P<.01, ***P<.001.

The conditional LGCM incorporated patient engagement as the time-varying covariate. It showed that an increased frequency of completed measurements was significantly linked to reduced systolic blood pressure at 3, 6, and 12 months. While not statistically significant, a comparable trend was observed at 9 months. The conditional model demonstrated good fit (Figure 4).

Here, age, sex, and comorbidity status were incorporated as covariates. The conditional LGCM indicated that neither sex nor comorbidity status significantly influenced the intercept or the slope of systolic blood pressure (Table 4). Age significantly influenced the intercept, suggesting that younger participants tended to have lower baseline systolic blood pressure. The time-varying variable of patient engagement significantly influenced blood pressure at 3, 6, and 12 months (frequency of completed measurements: β_3_=–0.016, *P*=.002; β_6_=–0.006, *P*=.02; β_12_=–0.003, *P*=.02). However, its effect on systolic blood pressure at 9 months was not statistically significant (β_9_=–0.002, *P*=.44). After adjusting for the underlying trajectory of systolic blood pressure and baseline characteristics, participants with a higher frequency of completed measurements reported lower systolic blood pressure at 3, 6, and 12 months.

**Table 4.** Estimated parameters for conditional latent growth curve model for systolic blood pressure (SBP; N*=*1723).

Covariate parameters	Estimate	SE	*P* value
Baseline characteristics			
Age→intercept	0.175	0.052	.001
Sex→intercept	−0.006	0.711	.99
Disease→intercept	1.191	1.429	.40
Age→slope	0.033	0.021	.12
Sex→slope	0.283	0.272	.30
Disease→slope	−0.444	0.544	.42
Measures of patient engagement			
Engagement_3_→SBP_3_	−0.016	0.005	.002
Engagement_6_→SBP_6_	−0.006	0.003	.02
Engagement_9_→SBP_9_	−0.002	0.003	.44
Engagement_12_→SBP_12_	−0.007	0.003	.02

## Discussion

### Principal Findings

The research is one of the first to examine the dynamic temporal relationship between patient engagement and blood pressure levels among older individuals with hypertension using longitudinal data from an mHealth intervention. We evaluate various aspects of this relationship by measuring patient engagement through the frequency of completed measurements. Our research examines the relationship between patient engagement and intervention benefits, providing insights into dose-response relationship and temporal features of mHealth interventions. The LGCM results indicate that patient engagement has a positive impact on blood pressure treatment effects, with this influence not only persisting throughout the 12-month research period but also increasing over time.

### Lessons Learned

First and foremost, current research offers novel insights on the dose-response relationship within mHealth interventions, an area where existing literature is limited and inconclusive [8,26]. There is a significant gap in the literature on current mHealth interventions regarding whether the momentum of intervention effects is sustained over time in long-term follow-up and whether patient engagement contributes to this momentum. The conflicting findings in previous mHealth intervention studies may be attributed to several factors, including improper study design or implementation, insufficient intervention duration, unclear targeting of the participant population, and inappropriate or inadequate measurement and statistical analysis methods for patient engagement [18,26,36,37]. There are some explanations as to why the dose-response relationship observed in this study is sustained and increased. Notably, interventions that are evidence-based, rigorously designed, and effectively implemented tend to yield sustained dose-response effects [38]. The study contributes to current literature by providing new evidence. It investigates a large cohort of older adults enrolled in an mHealth program, with up to 12 months of real-world follow-up data. Moreover, this study objectively examines the influence of patient engagement on the long-term effects of interventions using appropriate statistical analysis methods. This finding is significant as it highlights that mHealth interventions might exhibit diverse real-world correlations with health outcomes due to varying levels of patient engagement.

### Relationship Between Patient Engagement and Health Improvements

Identifying the ideal timing for the temporal relationship between patient engagement and health improvements is crucial. It enables better refinement of intervention design, pacing, and determining the appropriate duration. The unconditional LGCM results show that systolic blood pressure begins to decrease from the third month and follows a nonlinear pattern over the 12-month period. The steepest decline occurs at the ninth month compared to the initial point, after which the decrease slows down. Importantly, the decline at the 12th month remains greater than that at the sixth month. This pattern is similar to findings from another mHealth study targeting patients with heart failure, which demonstrated that the tangible benefits of mHealth interventions became prominent around 4‐5 months but plateaued after 8‐9 months, maintaining a stable trend [39]. The nonlinear pattern may result from 2 factors: the cumulative effect of patient engagement on health improvement and the time required for behavior change to take effect. Our analysis reveals that patient engagement peaks around the 9-month timepoint compared to the third and sixth months, indicating that patients’ willingness to participate in medical activities reaches its highest level at this stage. This suggests that the high-level patient engagement at 9 months may contribute to the observed pattern.

From the perspective of short-term mHealth research, studies show that interventions of insufficient duration may fail to deliver the desired results [40]. However, from the perspective of long-term mHealth studies, the observed decline after the ninth month does not necessarily indicate an end to the intervention’s benefits within the 1-year mHealth intervention. In fact, it implies that the positive effects may persist and even progress further, extending past this juncture. This continued benefit is particularly important for patients with frequent uncontrolled hypertension. For such individuals, it is essential to effectively manage blood pressure levels to prevent serious complications, including cardiovascular diseases. Therefore, subsequent interventions aimed at increasing patient engagement may be essential to maintain the momentum of these health improvements.

### Patient Engagement

Beyond identifying when dose–response relationships emerge within the intervention, it further explores potential time-varying associations between patient engagement and treatment effects. We used the frequency of completed measurements as a reliable measure of patient engagement to assess this association. The conditional LGCM results demonstrated that participants with higher patient engagement tended to observe reduced systolic blood pressure at the 3-, 6-, and 12-month intervals following baseline, after accounting for the underlying trajectory of systolic blood pressure and initial patient characteristics. To be specific, at the second timepoint of the intervention, participants had completed the measurements 32.3 times on average. Given that these recordings were made at 3-month intervals, this equates to a completion frequency of approximately once every 3 days (32.3 times/90 days). Our findings are consistent with previous findings in older individuals who also completed chronic disease management challenges every 3 days (10 times /30 days) achieved better cardiac assessment results [41]. Based on these frequency data, we infer that an ideal frequency of blood pressure monitoring once every 3 days may be beneficial for hypertension management. This is consistent with a recent study suggesting that 3 days of monitoring may be sufficient to determine or rule out elevated blood pressure in most patients [42]. The indicator captures a critical issue of patient engagement, reflecting the participants’ willingness to repeatedly engage with the intervention [26]. Furthermore, it indicates that adherence to this intervention can have clinically significant effects because it correlates patient engagement, quantified into clear participation thresholds, with subsequent improvements in blood pressure. This connection not only highlights the tangible benefits of consistent patient involvement but also reminds us of the importance of setting and achieving specific engagement targets in mHealth interventions. By doing so, health care providers can better adjust their programs to encourage patient participation and ultimately drive better health outcomes.

### Limitations

This study has several limitations. First, excluding patients who used the mHealth app for fewer than 12 months may introduce selection bias toward better adherence and treatment effects, limiting the findings’ generalizability. Second, the study is geographically limited to specific regions in Zhongshan City, China, which may affect the representation of the broader older hypertensive population due to regional differences in medical resources, culture, and patient behavior. Third, measuring patient engagement solely by the frequency of completed measurements overlooks its multidimensional nature, including interactions with health care providers and self-management plan implementation. Future research should incorporate these factors for a more comprehensive understanding. We plan to conduct multicenter studies with extended follow-up to better assess the mHealth intervention’s impact.

### Conclusions

In conclusion, our study demonstrates the positive impact of patient engagement on hypertension management in older adults through a 12-month mHealth intervention, using LGCMs and repeated measure data from 5 time points. The influence not only persisted throughout the 12-month research period but also increased over time. The results stress the importance of integrating patient-centered engagement approaches into mHealth programs designed for chronic disease management in aging populations.

## Abbreviations

HBPM: home blood pressure monitor
LGCM: latent growth curve model
mHealth: mobile health
